# Transcriptome analyses reveal photosynthesis-related genes involved in photosynthetic regulation under low temperature stress in *Lavandula angustifolia* Mill.

**DOI:** 10.3389/fpls.2023.1268666

**Published:** 2023-11-16

**Authors:** Ling Li, Yuchen Liang, Yinan Liu, Zeyi Sun, Yuning Liu, Zening Yuan, Chang Fu

**Affiliations:** ^1^Key Laboratory of Aquatic Biodiversity Research in Hei Longjiang Province, Harbin Normal University, Harbin, China; ^2^Heilongjiang Provincial Key Laboratory of Plant Biology in Ordinary Colleges and Universities, Harbin Normal University, Harbin, China; ^3^College of Science and Technology, Harbin Normal University, Harbin, China

**Keywords:** DEGs (differentially expressed genes), *Lavandula angustifolia* Mill., low temperature stress, photosynthetic regulation, qRT-PCR

## Abstract

In order to reveal the mechanisms of photosynthetic regulation of *Lavandula angustifolia* Mill. under low temperature stress, photosynthesis-related genes were screened and the molecular mechanism were analyzed for this species growing in Harbin, northeast of China. RNA-seq technique and photosynthetic physiology measurement were performed under 20°C, 10°C, and 0°C in this study. The results showed that the observing modified rectangular hyperbola mode could accurately reflect the light-response processes under low temperature stress and the low temperature reduced the light energy utilization of *L. angustifolia*. The stomatal conductance decreased with the temperature dropping, which was associated with the up-regulation of *LaBAM1s*, *LaMPK4-1* and *LaMMK2*. The up-regulation of *LaMPK4-1* and *LaMMK2* was beneficial for ROS scavenging. The improvement of cold resistance in *L. angustifolia* was related to the up-regulated expression of *LaFBA* and *LaOMTs* and down-regulated expression of *LaGAPAs*, *LaGOX*, and *LaTKL1s* with the temperature decreasing. The up-expression of *LaPSY* at 10°C than it at 20°C could protect the photosynthetic organs from oxidative damage. Moreover, the photosynthetic rates at 10°C and 0°C were close to the measured values, which was related to the interactions of RCA with SBPase and Rubisco with SBPase. These findings could provide a theoretical reference for further exploring the cold tolerance mechanism of *L. angustifolia*, as an important aromatic plant resource, and promoting its cultivation and distribution in the northeast of China.

## 1 Introduction

Photosynthesis, as the most crucial process of physiological and biochemical reaction on Earth, is the basis for plant growth and development and also for high yield and quality. More than ninety percent of the plant’s dry weight is derived from leaf photosynthetic products (Yang et al., 2022). Photosynthesis is the first damaged physiological process inhibited by low temperature stress which is the first line constraint that obstructs the growth, development, and yield potential of plants (Zhang et al., 2020). Low temperature could lead to photosynthesis inhibition and transpiration rate reduction (Zhang et al., 2023). And the direct cause of the decline in photosynthetic efficiency is \stomatal closure (Amin et al., 2023). The light response curves of photosynthesis can characterize the variations in photosynthetic rate and estimate the main photosynthetic parameters, such as apparent quantum yield (AQY), maximum net photosynthetic rate (*P_nmax_*), light saturation point (*LSP*), light compensation point (*LCP*), and dark respiration rate (*R_d_*), etc. These indices could reflect the photosynthetic physiological and ecological regulation of plants in response to adversity and could be beneficial in analyzing the function of the photosynthetic apparatus, capacity, and efficiency of plants and the extent to which they are affected by environmental changes (Lang et al., 2011). Among these indices, *R_d_* is significantly influenced by temperature (Grisafi and Tombesi, 2023).

Some ways in which low temperature interferes with photosynthesis mainly including the disruption of the photosynthetic pigment complex system and the membrane fluidity, the reduction of the photochemical efficiency of plant photosystems (PSI and PSII), the decrease in enzymatic activities, and the changes in electron transfer rate in thylakoid and metabolic processes (Özdemir et al., 2023; Xu et al., 2023). Moreover, low temperature brought photodamage of the PSII reaction center (Velitchkova et al., 2023), altered the light-harvesting antenna system and reaction center structure, disrupted the photosynthetic structure (Sachdev et al., 2023), and caused inhibition of photosynthesis (Zhang et al., 2023), which decreased the photosynthetic capacity (Guo et al., 2023). However, PSII can adjust the photosynthetic rate under abiotic stress because the involvement of PsbQ (PSII oxygen-evolving enhancer protein 3) would be conducive to the mediation of PSII assembly or stability (Bricker and Frankel, 2011; Guo et al., 2023).

Low temperature stress reduced light absorption efficiency of photosystems (PSI and PSII) and impaired electron transfer owing to the decline of pigment content (Bhattacharya, 2022). Studies have shown that *CHLD* (*magnesium chelatase D subunit*), *HEMA* (*glutamyl-tRNA reductase*), and *PORA* (*protochlorophyllide oxidoreductase*) genes are required for chlorophyll biosynthesis in *Arabidopsis thaliana*. *PSY* (*phytoene synthase*) is an optimal candidate gene for understanding the molecular regulation of carotenoid accumulation in most species (Ezquerro et al., 2023). Carotenoids, as auxiliary light-trapping pigments (Du et al., 2019), are involved in the dissipation of excess light energy in the photosynthetic mechanism of plants, reduce plant damage caused by photoinhibition, and protect the photosynthetic apparatus against photo-oxidative damage (Wang et al., 2020). Low temperature stress causes photodamage and hinders photosynthetic electron transfer in coconut (*Cocos nucifera*), tea (*Camellia sinensis*), and watermelon (*Citrullus lanatus*) under the conditions of the down-regulated expression of *FD1* (*ferredoxins*)and *FNR* (*ferredoxin-NADP^+^ reductase*) genes (Li et al., 2018; Shi et al., 2019; Yang et al., 2020), ultimately affecting the formation and accumulation of photosynthetic product (Wu et al., 2014). However, down-regulated expression of *TKL* (*transketolase*), *GAPA* (*glyceraldehyde-3-phosphate dehydrogenase*), and *GOX* (*glycolate oxidase*) genes (Yang et al., 2006; Chen et al., 2019) and up-regulated expression of *FBA* (*fructose-bisphosphate aldolase*), *BAM1* (*β-amylase*), and *BAM3* (*β-amylase*) genes (Ma et al., 2022; Qiu et al., 2023; Wang et al., 2023) could co-regulate to increase the content of soluble sugar and thus improve the resistance when plants were exposed to low temperature environments (Fulton et al., 2008). In addition, the storage forms of sugar can be catalyzed by β-glucosidases (BGLUs) into different complexes that sustain plant physiology stability (Chen L. et al., 2023). Among these genes, starch is rapidly mobilized by the synergistic action of *BAM1* and *BAM3* to promote stomatal opening (Thalmann et al., 2016).

*Lavandula angustifolia* Mill. is an important aromatic plant resource, with the main uses of ornamental, medicinal, and health care, etc. (Gregory, 2021; Neul et al., 2022; Seiiedi-Biarag and Mirghafourvand, 2022). Currently, studies on the effects of abiotic stress on photosynthesis in lavender have mainly focused on drought stress which inhibited the growth and decreased the photosynthetic pigments (Szekely-Varga et al., 2020), providing a theoretical basis for the introduction and industrialization of lavender in stress environment. The optimum temperature for lavender growth usually ranges from 15°C to 30°C (Zhang, 2020). Our previous studies have shown that low temperature stress (0°C) could activate the expression of functional genes related to synthesizing protective substances such as fatty acid desaturases, soluble sugars, and other protective substances, which formed a cold signaling regulatory network, consequently improving *L. angustifolia* cold tolerance (Lin et al., 2022; Lin et al., 2023). Also, research on the molecular regulation of photosynthesis coping with low temperature stress would be helpful in revealing the mechanisms of adaptation to low temperatures and promoting this species spread in northern alpine regions. Therefore, transcriptome sequencing technology was performed combing with bioinformatics methods and photosynthetic physiological characteristics were investigated for *L. angustifolia* under low temperature stress in this research. Leaf photosynthesis-related DEGs and key genes involved in photosynthesis regulation were also analyzed.

The aims of the study are as follows: 1) To investigate the responsive characteristics of photosynthetic physiology and to explain the applicability of different light response models to fit the light response process of *L. angustifolia* in response to low temperature 2) To mine key photosynthetic genes related to the regulation of photosynthesis under low temperature stress and analyze the function of genes to provide candidate genes for improving photosynthetic efficiency in *L. angustifolia.* 3) To establish the regulatory network of key photosynthesis genes in response to low temperature and to lay a scientific foundation for molecular breeding of *L. angustifolia* for cold tolerance.

## 2 Materials and methods

### 2.1 Plant materials and growth conditions

We transplanted two-year-old *L. angustifolia* grown in Harbin Normal University (Harbin, Heilongjiang, China), and then selected *L. angustifolia* plants with similar health and growth potential for cultivation. Plants were grown in an illuminated incubator (RGX250E, Tianjin, China) at a photoperiod 12h/12h (day/night) and the initial measurements were made at 20°C (control), and then temperatures were reduced at a rate of 2°C/h to about 10°C and 0°C.

Three replications were set up for each temperature. Leaf tissues (0.5g) were collected and quickly soaked in liquid nitrogen and then stored in a freezer (-80°C) after being cultured at each temperature for 24 h for the subsequent assays of transcriptome sequencing. The leaf tissues were acquired from the same replicate so that the materials definitely came from the same clone under different temperatures.

### 2.2 Measurement of photosynthetic response process and its simulation in *L. angustifolia* leaves

Data for analyzing photoresponse model was also determined under each temperature with 5 plants as one duplication, 2 similar potential growth leaves, and 3 different positions on each leaf per plant, for one dataset measuring 30 times.

To measure the net photosynthetic rate (*P_n_*) under the condition of photosynthetically active radiation (PAR), we used a portable LS-1020 gas exchange system (Hangzhou, China) equipped with a red/blue light-emitting diode (LED) light source, which can adjust the PAR from 1 800 *μ*mol·m^-2^·s^-1^ reducing to 1.500, 1.200, 900, 600, 400, 200, 150, 100, 50, 30, and 0 *μ*mol·m^-2^·s^-1^. The values of *P_n_* were automatically recorded by the portable gas exchange system, and the mean values were taken for analysis. Measurements were made at mean leaf temperature of 22.9°C ± 0.3˚C, and a vapor pressure deficit (VPD) of 1.01 ± 0.1 kPa.

The software (Photosynthetic Computing 4.1.1) was used to obtain the light response curves fitted by the modified rectangular hyperbola mode (MRH), the rectangular hyperbola model (RH), the non-rectangular hyperbola model (NRH), and the exponential model (EXP) (Lang et al., 2013).

The fitted values of *P_n_*, maximum net photosynthetic rate (*P_n_*_max_), light saturation point (*LSP*), light compensation point (*LCP*), and dark respiration rate (*R_d_*) were calculated from each model. The fitting effects of the models were evaluated with the reference of mean squared error (*MSE*), mean absolute error (*MAE*), and *R^2^* (Yuan et al., 2022).

The photosynthetic physiological data were collated with Excel (2301 Build 16.0.16026.20002), analyzed with SPSS 25.0, and plotted with Origin 2022.

### 2.3 Measurement of photosynthetic gas exchange parameters and photosynthetic pigments in *L. angustifolia* leaves

The photosynthetic gas exchange parameters were measured via LS-1020 photosynthesis gas exchange system with 3 plants as one replication, 3 similar potential growth leaves, and 3 adjacent positions on each leaf per plant, for one dataset testing 27 times.

A portable LS-1020 photosynthesis gas exchange system equipped with a red/blue light-emitting diode (LED) light source was used for the PAR 1 500 *μ*mol·m^-2^·s^-1^, measuring the photosynthetic indexes including photosynthetic rate (*P_n_*), transpiration rate (*T_r_*), stomatal conductance (*G_s_*), intercellular CO_2_ concentration (*C_i_*), external CO_2_ concentration (*C_a_*), and water use efficiency (WUE). Stomatal limit value (*L_s_*) was calculated according to the formula *L_s_=*1-*C_i_*/*C_a_*. Measurements were made at mean leaf temperature of 22.9°C ± 0.3˚C, and a vapor pressure deficit (VPD) of 1.01 ± 0.1 kPa.

After the measurements of photosynthesis gas exchange, chlorophyll and carotenoids (*Car*) contents were determined in a whole-pigment extract of leaf tissues by UV-VIS spectroscopy. The absorbance of the extract was measured at 447 nm, 665 nm, and 649 nm for the calculation of the chlorophyll *a* (*Chl a*), chlorophyll *b* (*Chl b*), and *Car* contents, respectively. The concentrations of *Chl a*, *Chl b*, chloroplast pigment (*Chl*), chlorophyll *a+b* (*Chl a+b*), chlorophyll *a/b* (*Chl a/b*), and *Car* were calculated using the following formulas.


$$Chl\;a\;\left(\text{mg}/\text{L}\right)=13.95{\text{A}}_{665}-6.88{\text{A}}_{649}$$

$$Chl\;b\;\left(\text{mg}/\text{L}\right)=24.96{\text{A}}_{649}-7.32{\text{A}}_{665}$$

$$Car\;\left(\text{mg}/\text{L}\right)=\left(1000{\text{A}}_{470}-2.05{\text{C}}_{\text{a}}-114.8{\text{C}}_{\text{b}}\right)/245$$

Chl (mg/g)=(C·V·N)/(m ·1000)

### 2.4 Sequencing of the transcriptome in *L. angustifolia* leaves

Leaf tissues collected in 2.1 were taken to transcriptome analysis, and three replicates were analyzed for each temperature. The total RNA of 9 *L. angustifolia* samples was extracted from leaf tissues using a TRIzol reagent to ensure the use of qualified samples for transcriptome sequencing. Beijing Ruibo Xingke Biotechnology Co., Ltd. was entrusted to conduct transcriptome sequencing using the Illumina Hi Seq 2 500 platform and 2.3 GB of transcriptome data per sample were generated.

The raw data were submitted to the SRA database (https://www.ncbi.nlm.nih.gov/sra/) of NCBI (https://www.ncbi.nlm.nih.gov/) with the login numbers PRJNA765132. After filtering and screening the raw data, high quality Clean Reads were obtained. Then, differentially expressed genes (DEGs) were analyzed and compared with the database for predictive analysis of the classification, function, and enrichment of related genes in metabolic pathways on the conditions of 0°C vs 10°C, 10°C vs 20°C, and 0°C vs 20°C.

### 2.5 Analysis of photosynthesis-related DEGs in *L. angustifolia* leaves

Photosynthesis-related DEGs were analyzed and heat maps were generated through TBtools v1.086. These DEGs were classified according to different functions, and the correlation analysis and network diagram between the classified DEGs and photosynthetic physiological parameters were performed using an online platform (http://www.cloud.biomicroclass.com/).

Predictive analysis of conserved motifs of amino acid sequences concerning photosynthesis-related DEGs was performed using the online website (https://www.ncbi.nlm.nih.gov/Structure/cdd/wrpsb.cgi), and visualization of conserved structural domains was conducted by TBtools software using CDD hitdata files obtained from the NCBI.

The online tools ExPASy (https://web.expasy.org/protparam/) and WoLF PSORT (https://www.genscript.com/tools/wolf-psort) were employed to explore basic physicochemical properties of amino acid sequences and predict subcellular localization.

The tertiary structure of the protein was predicted by PhysE2 and tertiary images were generated by VMD 1.9.2 software. The STRING protein interactions database (http://string-db.org/) was applied to compare the assembled sequences with protein sequences of *A. thaliana*, visualize the network editing of gene properties according to the target genes, and produce protein network interaction maps.

### 2.6 Screening and validation of DEGs for photosynthesis in *L. angustifolia* leaves

The genes involved in the composition of photosynthetic apparatus and light energy transmission were selected for qRT-PCR analysis. Plant total RNA was isolated using a Minibest plant RNA extraction kit (Takara, China). cDNA synthesis and qRT-PCR analyses was performed with Primescript RT reagent kit with gDNA eraser (Takara, China) on QuantStudio™3 (Applied biosystems, USA). The primers were designed in Primer 3.0 software and the pair of primers are presented in **Table 1**. *Gapdh* was used as the reference gene. The 2^−ΔΔCT^ method was used to calculate the relative expression levels of the target genes. All reactions were performed with three biological and technical replicates.

**Table 1:** Primers used for qRT-PCR in *L. angustifolia* leaves.

NO.	Gene Name	Sequence of primer(5′-3′)	Melting Temperature(Tm)
1	*LaBAM1-3*	F-CGGAAGAAGGCGATGAAC	56.0°C
		R-CGCACTGGTGAAACGACA	56.1°C
2	*LaCHLG*	F-CTACCCAACTCTGTCTACTCAC	54.2°C
		R-GACATCTTCCACAGTCCAGT	54.1°C
3	*LaFD-1*	F-CTTTATCTCCGAAGACCCTC	51.9°C
		R-TCATCATACGCTTGTGGC	52.4°C
4	*LaMPK4-1*	F-GTCCAACCAAACACTAACCG	53.9°C
		R-GCCCGAAGTCTCCGATT	54.9°C
5	*LaPSAA-1*	F-CAAGTTTTGCCGAAGGAG	55.8°C
		R-CCCGCTATCAGGAACAGA	55.6°C
6	*LaPSBA-1*	F-CTTCATTGCTGCTCCTCC	53.5°C
		R-ACTAAGTTCCCACTCACGAC	54.0°C
7	*LaPSBQ*	F-TCGGAGAAGAGGCAGAAT	52.5°C
		R-CGCAGATACCTCCTAACG	52.3°C
8	*LaTKL1-3*	F-AGGTTTGGTGTCCGTGAA	53.9°C
		R-TTGGCTGATGGGTAGGTC	54.3°C
9	GAPDH	F-TAGGAGGTGGCAGGACATCA	58.0°C
		R-CCCTTTACCCGTCACGTTGT	57.8°C

The primers were synthesized by Beijing Rui Bo Xing Ke Biotechnology Co. The experimental data were analyzed to more deeply verify the reliability of the sequencing data by GraphPad Prism 8.0.1.

## 3 Results and analysis

### 3.1 Photosynthetic physiological characteristics in *L. angustifolia* leaves

#### 3.1.1 Analysis of the light response curve models

**Figure 1:**
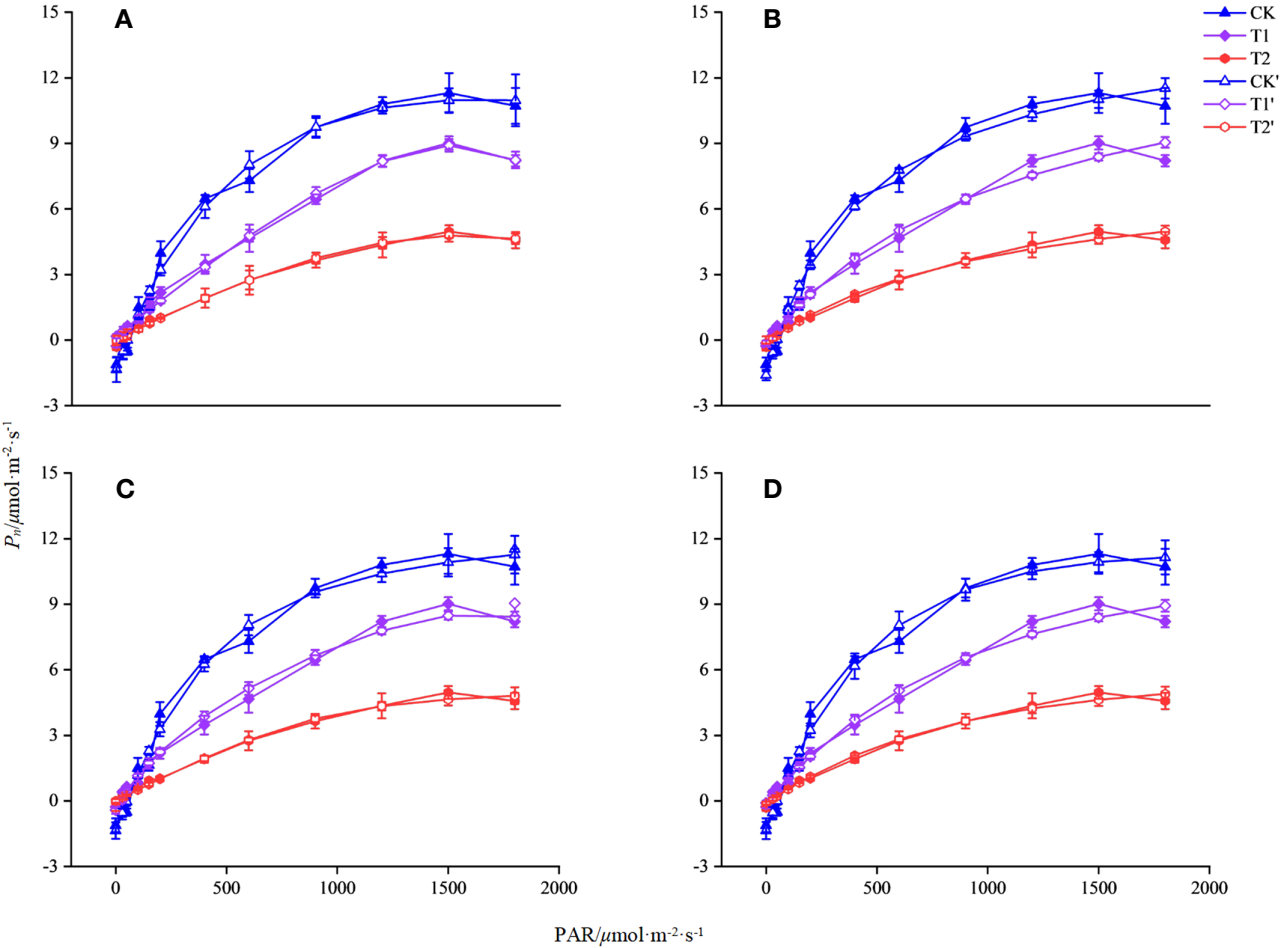
Simulation of photosynthetic light response curve of *L. angustifolia* by different light response models under different temperatures. CK, T1, and T2 represent measured value curves at 20°C, 10°C, and 0°C, respectively; CK’, T1’, and T2’, represent the fitting model curves at 20°C, 10°C, and 0°C, respectively. **(A–D)** are the modified rectangular hyperbola mode (MRH), the rectangular hyperbola model (RH), the non-rectangular hyperbola model (NRH), and the exponential model (EXP), respectively (n=30). Values=M ± S.E.

In this study, four models of MRH, RH, NRH, and EXP were used to analyzed the characteristic of the light response for *L. angustifolia* under different temperatures. It can be estimated from the four models that 1 500 *μ*mol·m^-2^·s^-1^ was the *LSP* of *L. angustifolia* leaves. Among these models, the MRH showed an increasing and then decreasing trend, which was consistent with the measured values with the enhancement of PAR under different temperatures (**Figure 1A**). The remaining three models did not exhibit better simulation between estimated and measured values (**Figures 1B–D**).

The *R^2^* of the MRH was higher than that of the other three models, with the smallest *MAE* and *MSE* under different temperatures. It is known that the closer *R^2^* is to 1, the better the correlation is presented, with the smaller values of *MAE* and *MSE* implying that the MRH was the best linear model (**Table 2**).

**Table 2:** Comparison of the fitted values of light response parameters between measured and simulated in *L. angustifolia* under different temperatures.

Light Response Model	Treatment Temperature	Light Response Parameters									
		AQY/ (mol/mol)			*LSP*/ (*μ*mol·m^-2^·s^-1^)	*LCP*/ (*μ*mol·m^-2^·s^-1^)	*P_n_*_max_/ (*μ*mol·m^-2^·s^-1^)	*R_d_*/ (*μ*mol·m^-2^·s^-1^)	*R*^2^	*MSE*	*MAE*
		*Φ_c_*	*Φ_0_*	*Φ_c0_*							
Actual Data	20°C	–	–	–	≈1500	45.09	11.31	-1.01	–	–	–
	10°C	–	–	–	≈1500	0.42	9.02	-0.27	–	–	–
	0°C	–	–	–	≈1500	12.96	4.97	-0.31	–	–	–
MRH	20°C	0.024	0.027	0.026	1555.30	54.86	11.01	1.40	0.99	0.23	0.43
	10°C	0.009	0.008	0.008	1540.90	-21.69	8.91	-0.18	0.99	0.06	0.21
	0°C	0.005	0.005	0.005	996.58	4.35	4.79	-0.02	1.00	0.02	0.12
RH	20°C	0.029	0.035	0.032	764.42	52.85	11.59	1.68	0.98	0.31	0.54
	10°C	0.013	0.013	0.013	1435.01	11.02	9.05	0.14	0.98	0.19	0.37
	0°C	0.007	0.008	0.008	1280.95	23.16	4.98	0.18	0.99	0.04	0.18
NRH	20°C	0.018	0.025	0.025	562.21	56.67	11.23	1.39	0.98	0.26	0.49
	10°C	0.010	0.016	0.015	695.54	14.65	9.33	-0.22	0.97	0.13	0.34
	0°C	0.000	0.005	0.005	996.62	4.01	4.81	0.02	0.99	0.03	0.15
EXP	20°C	0.023	0.025	0.024	1803.83	47.46	11.19	1.13	0.99	0.24	0.47
	10°C	0.011	0.012	0.012	1795.00	90.38	8.93	1.01	0.98	0.16	0.37
	0°C	0.006	0.007	0.006	820.81	167.55	4.91	1.03	0.99	0.04	0.17

MRH, the modified rectangular hyperbola mode; RH, the rectangular hyperbola model; NRH, the non-rectangular hyperbola model; EXP, the exponential model. AQY, Apparent Quantum Yield; LSP, Light Saturation Point; LCP, Light Compensation Point; P_n__max_, Maximum Net Photosynthetic Rate; R_d_, Dark Respiration Rate; MSE, Mean Squared Error; MAE, Mean Absolute Error.

The MRH was suitable for simulating the response process of *P_n_-*PAR and estimating the photosynthetic parameters of *L. angustifolia* at low temperature (**Figure 1**; **Table 2**). From the MRH, *LCP* and *R_d_* were observed to decline at 10°C compared to 20°C, but increase at 0°C compared to 10°C (**Table 2**).

#### 3.1.2 Analysis of photosynthetic gas exchange parameters and photosynthetic pigment

*G_s_* and *T_r_* decreased with decreasing temperature, the maximum value of *L_s_* was observed at 20°C and the minimum value at 10°C. These parameters showed no significant differences between 20°C, 10°C, and 0°C (*p*<0.05) (**Table 3**). Values of *C_i_*, *C_a_*, and WUE were the minimum at 20°C and the maximum at 10°C, while values of *C_i_* and *C_a_* were significantly higher at 0°C and 10°C than those at 20°C(*p*<0.05) (**Table 3**).

**Table 3:** Comparison of photosynthetic gas exchange parameters and photosynthetic pigments of *L. angustifolia* leaves under different temperatures.

Parameters	20°C	10°C	0°C
*G*s(×10-3 mol.m-2.s-1)	8.43 ± 0.50a	6.70 ± 1.54a	4.97 ± 1.90a
*T*r(×10-2 mmol.m-2.s-1)	11.81 ± 0.84a	11.28 ± 3.81a	7.62 ± 2.80a
*L*s*(*×10-3)	5.70 ± 1.48a	3.30 ± 0.55a	6.70 ± 2.09a
*C*i(×102 ppm)	5.90 ± 0.24b	8.08 ± 0.42a	7.71 ± 0.27a
*C*a(×102 mmol)	5.94 ± 0.24b	8.11 ± 0.43a	7.91 ± 0.26a
WUE(×10 mmol.mol-1)	9.90 ± 0.85a	12.00 ± 4.65a	10.30 ± 3.38a
*Chl a*(mg/L)	22.09 ± 1.02a	20.70 ± 0.70a	15.19 ± 0.47b
*Chl b*(mg/L)	7.95 ± 0.86a	7.95 ± 0.84a	5.25 ± 0.45b
*Car*(mg/L)	4.96 ± 0.55a	5.32 ± 0.18ab	4.14 ± 0.19b
*Chl*(mg/g)	0.90 ± 0.06a	0.86 ± 0.03a	0.61 ± 0.02b
*Chl (a+b)*(mg/L)	30.04 ± 1.85a	28.65 ± 0.84a	20.44 ± 0.81b
*Chl (a/b)*(mg/L)	2.96 ± 0.21a	2.88 ± 0.35a	3.05 ± 0.24a

Different letters in the same row indicate significant differences (p<0.05), the order of letters is based on the mean from largest to smallest. M ± S.E.(n=27).

Gs, Stomatal Conductance; Tr, Transpiration Rate; Ls, Stomatal Limit Value; Ci, Intercellular CO_2_ Concentration; Ca, External CO_2_ concentration; WUE, Water Use Efficiency; Chl a, Chlorophyll a; Chl b, Chlorophyll b; Car, Carotenoid; Chl, Chloroplast Pigment; Chl (a+b), Chlorophyll a+b; Chl (a/b), Chlorophyll a/b.

As shown in **Table 3**, the values of *Chl a*, *Chl b*, *Chl*, and *Chl (a+b)* of *L. angustifolia* leaves gradually decreased with the temperature declining. *Chl a/b* did not show significant differences under 20°C, 10°C and 0°C (*p*<0.05). All photosynthetic pigments were significantly reduced at 0°C compared to 10°C, except *Car* (*p*<0.05). *Chl b* decreased the most by about 34% and *Car* decreased the least by about 17% at 0°C compared to 20°C (**Table 3**).

### 3.2 Results of gene annotation and expression analysis of photosynthesis-related DEGs in *L. angustifolia* leaves

The transcriptomes of *L. angustifolia* leaves exposed to three temperature gradients (20, 10, and 0°C) was sequenced and the total assembled potential transcripts were 369 926 733 with GC contents of 41.62% and an N50 length of 1 578 bp, indicating a high level of assembly integrity. Clean reads rate and raw Q30 bases rate were higher than 90% at 20°C, 10°C, and 0°C, which showed better sequencing quality and provided good raw data for the subsequent data analysis (**Table 4**).

**Table 4:** Statistical analysis of filtered transcript data in *L. angustifolia* leaves.

Sample	Clean Reads Number	Clean Reads Rate (%)	Clean Bases Number	Ns Reads Rate(%)	Adapter Polluted Reads Rate(%)	Raw Q30 Bases Rate(%)	Clean Q30 Bases Rate(%)
20°C-1	43,624,252	95.73	6,543,637,800	0.18	3.56	92.98	93.25
20°C-2	46,245,032	94.45	6,936,754,800	0.21	4.89	93.34	93.61
20°C-3	44,757,118	96.26	6,713,567,700	0.18	3.09	93.63	93.9
10°C-1	46,311,568	92.47	6,946,735,200	0.26	6.87	93.64	93.96
10°C-2	45,423,838	93.76	6,813,575,700	0.19	5.63	93.61	93.92
10°C-3	46,514,604	93.45	6,977,190,600	0.24	5.94	93.6	93.88
0°C-1	44,072,738	95.32	6,610,910,700	0.21	4.04	93.74	94.01
0°C-2	46,481,702	94.66	6,972,255,300	0.27	4.62	93.45	93.78
0°C-3	45,884,320	92.67	6,882,648,000	0.36	6.52	93.73	94.09

1, 2, and 3 represent the number of sample replications, respectively.

**Figure 2:**
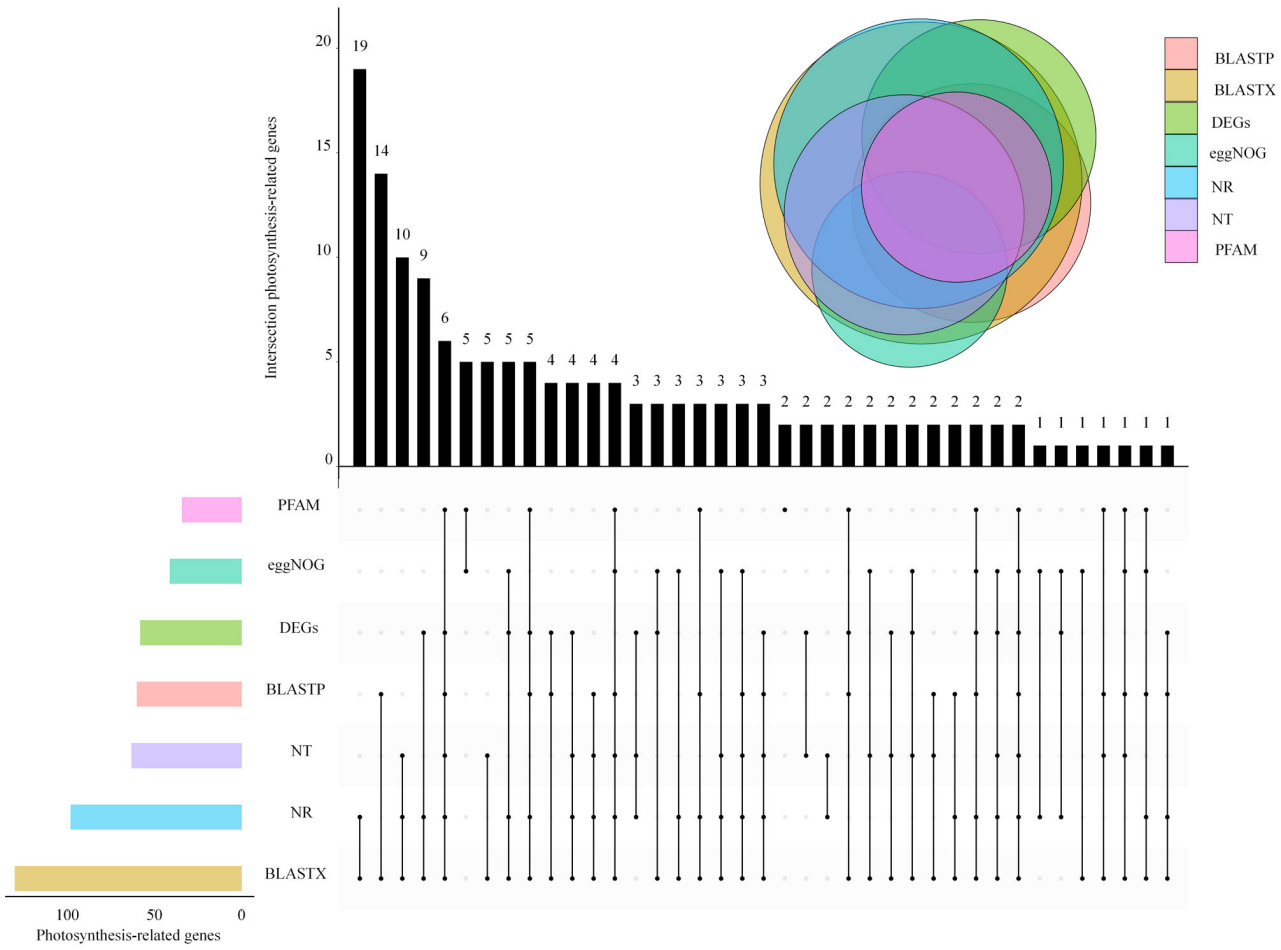
Venn diagram of photosynthesis-related expressed genes in *L. angustifolia* leaves.

By analyzing transcriptome data, 81 017 unigenes were compared with NR, NT, UniProt (BLASTX, BLASTP), PFAM, and eggNOG databases, and 146 unigenes were found related to photosystem. The maximum number of photosystem-related genes was 19 in BLASTX and NR databases. The enrichment of photosystem-related DEGs was different in every database, with the most abundant DEGs in the BLASTX and NR libraries (**Figure 2**).

**Figure 3:**
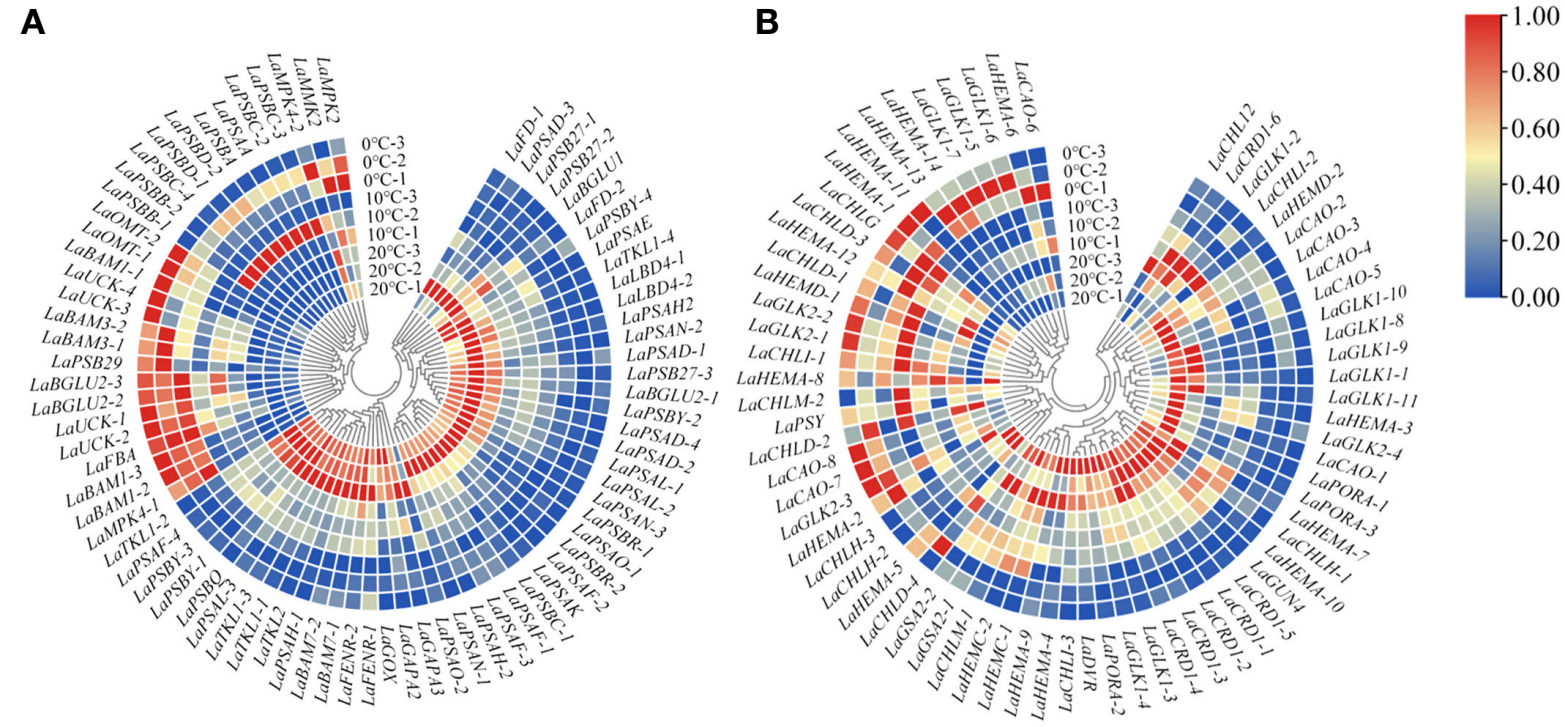
Quantitative heat maps of photosynthesis-related DEGs expression in *L. angustifolia* leaves. **(A)** Heat map of DEGs expression related to photosynthetic process; **(B)** Heat map of DEGs expression related to photosynthetic pigment.

Based on the changes of photosynthetic physiological characteristics, 79 photosystem-related DEGs were selected to shape the heat map (**Figure 3A**). It was found that the genes *LaBAM1-1*, *2*, *3*, *LaBAM3-1*, *2*, *LaFBA*, *LaOMT-1*, *2*(*O-methyl transferase*), and *LaUCK-1*, *2*, *3*, *4* for regulating carbon metabolism-related enzymes were up-regulated with decreasing temperature, while *LaFD-1*, *2*, *LaFENR-1*,*2*, *LaGAPA-2*, *3*, *LaGOX* (*glycolate oxidase*), and *LaTKL1-1*, *2*, *3*, *4* were down-regulated (**Figure 3A**).

Compared to 20°C, *LaMPK4-1*, *2* (*MAP kinase*) and *LAMMK2* (*MAP kinase kinase*) were down-regulated at 10°C, but up-regulated at 0°C (**Figure 3A**). With decreasing temperature, *LaPSAA-1* (*PSI protein*) in PSI and *LaPSBA* (*PSII D1 protein*), *LaPSBB-1*, *2* (*PSII CP47 chlorophyll apoprotein*), *LaPSBC-2*, *3*, *4* (*PSII protein CP43 chlorophyll apoprotein*), *LaPSBD-1*, *2* (*PSII D2 protein*) were up-regulated, but *LaPSBQ*, *LaPSB27-1*, *2*, *3* (*PSII repair protein Psb27-H1*) in PSII, and *LaLBD4-1*, *2* (*lateral organ boundary domain*) in LHCII were down-regulated (**Figure 3A**).

Considering the role of chlorophyll molecules in the antenna system collecting light energy and driving electron transfer from the reaction center, we screened 72 DEGs associated with photosynthetic pigment synthesis from the transcriptome data to generate a heat map (**Figure 3B**). Among these genes, *LaCHLG* (*chlorophyll synthase*) and *LaGLK2-1*, *2*, *3* (*golden 2-like*) were up-regulated, while *LaCHLH-1*, *2*, *3* (*magnesium chelatase H subunit*), *LaGLK1-1*, *2*, *3*, *4*, *8*, *9*, *10*, *11*, *LaGUN4* (*required for efficient Mg-chelatase activity*), and *LaPORA-1*, *2*, *3* were down-regulated with the decrease of temperature. At 10°C, *LaCHL12* (*magnesium chelatase I subunit*) and *LaPSY* presented the highest expression, but these two genes had the lowest expression at 0°C and 20°C, respectively (**Figure 3B**).

### 3.3 Photosynthesis-related protein network interactions in *L. angustifolia* leaves

**Figure 4:**
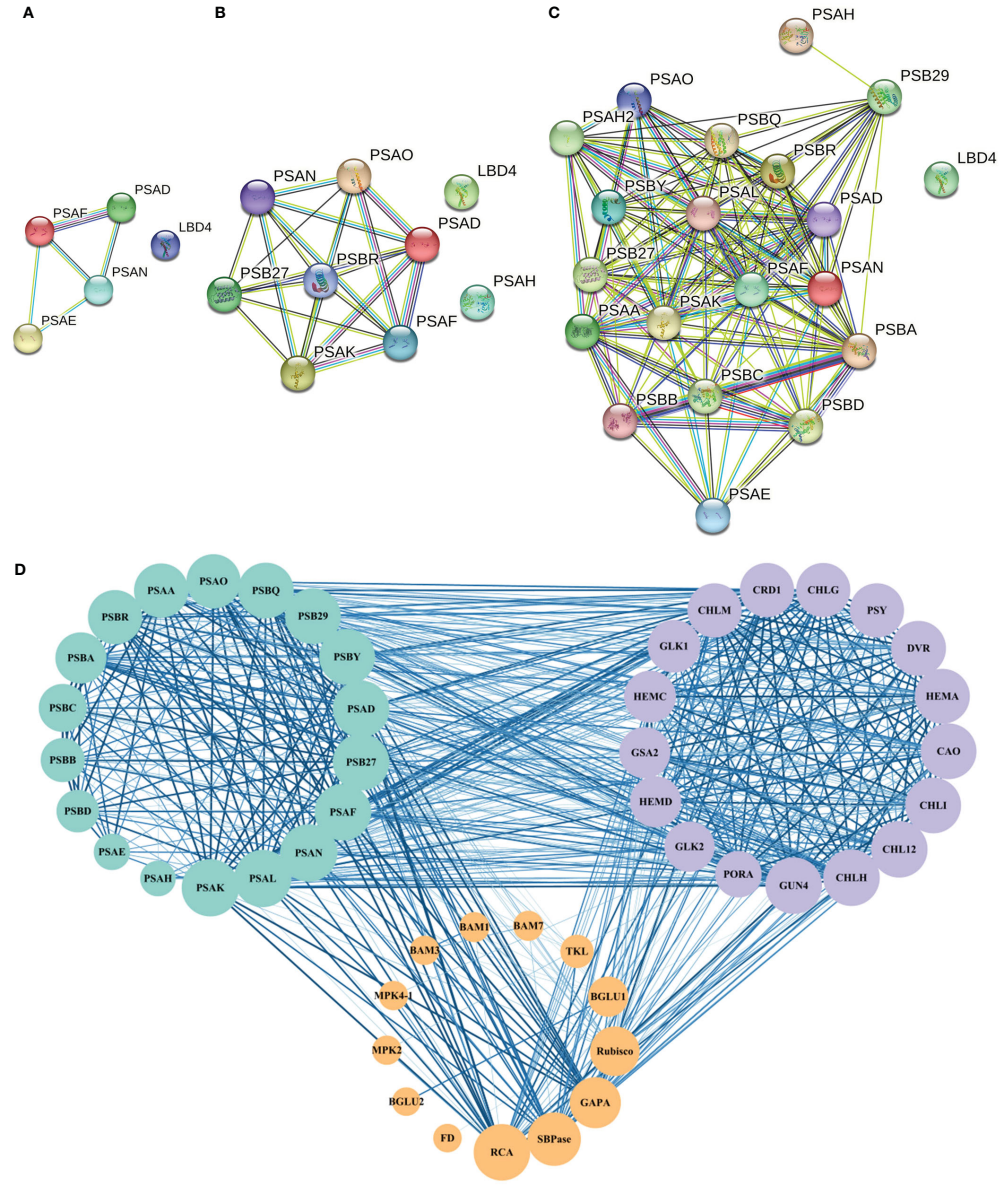
Protein interaction network of photosynthesis-related pathways in *A. thaliana.* **(A–C)** respectively represent the interaction of proteins related to the photosynthetic process at 0°C vs 10°C, 10°C vs 20°C, and 0°C vs 20°C. Nodes represent proteins, different colors represent different proteins, and the three-dimensional structure of protein is in the circle. The straight lines represent the interactions between proteins; **(D)** The protein network interaction among the photosynthesis process related proteins, photosynthesis related enzymes, and photosynthetic pigment synthesis related proteins. The size of the node represents the number of protein nodes that interacted with itself. A larger node represents more protein nodes interact with itself, and vice versa. The thickness and color depth of lines represent the strength of protein-protein interaction. The thicker and darker the lines are, the stronger the protein-protein interaction would be. On the contrary, the weaker the lines are.

Protein interaction network analysis was performed for the proteins regulated by DEGs that were related to photosynthesis using a database of *A. thaliana*. When 0°C vs 10°C, there were 4 photosynthetic process-related proteins interacting with each other (**Figure 4A**). When 10°C vs 20°C, 7 photosynthetic process-related proteins interacted with each other (**Figure 4B**). When 0°C vs 20°C, there had 19 photosynthetic process-related proteins interacting with each other (**Figure 4C**). The results showed that the interactions of proteins involved in photosynthetic process-related proteins became more complex under the difference of 0°C vs 10°C, 10°C vs 20°C, and 0°C vs 20°C (**Figures 4A–C**). For 0°C vs 20°C, the numbers of photosynthetic process-related proteins were the most (**Figure 4C**).

Based on the above results, interactions existed among the screened photosynthesis-related proteins (**Figure 4D**). Among the enzymes related to photosynthetic carbon metabolism, MPK4 and MMK2, BAM1 and BAM3, Rubisco (ribulose-1,5-bisphosphate carboxylase/oxygenase) and SBPase (scenic heptulose-1,7-bisphosphatase), and SBPase and RCA (Rubisco activase) all formed reciprocal relationships (**Figure 4D**).

### 3.4 Correlation between photosynthesis-related DEGs and photosynthetic physiological parameters in *L. angustifolia* leaves

**Figure 5:**
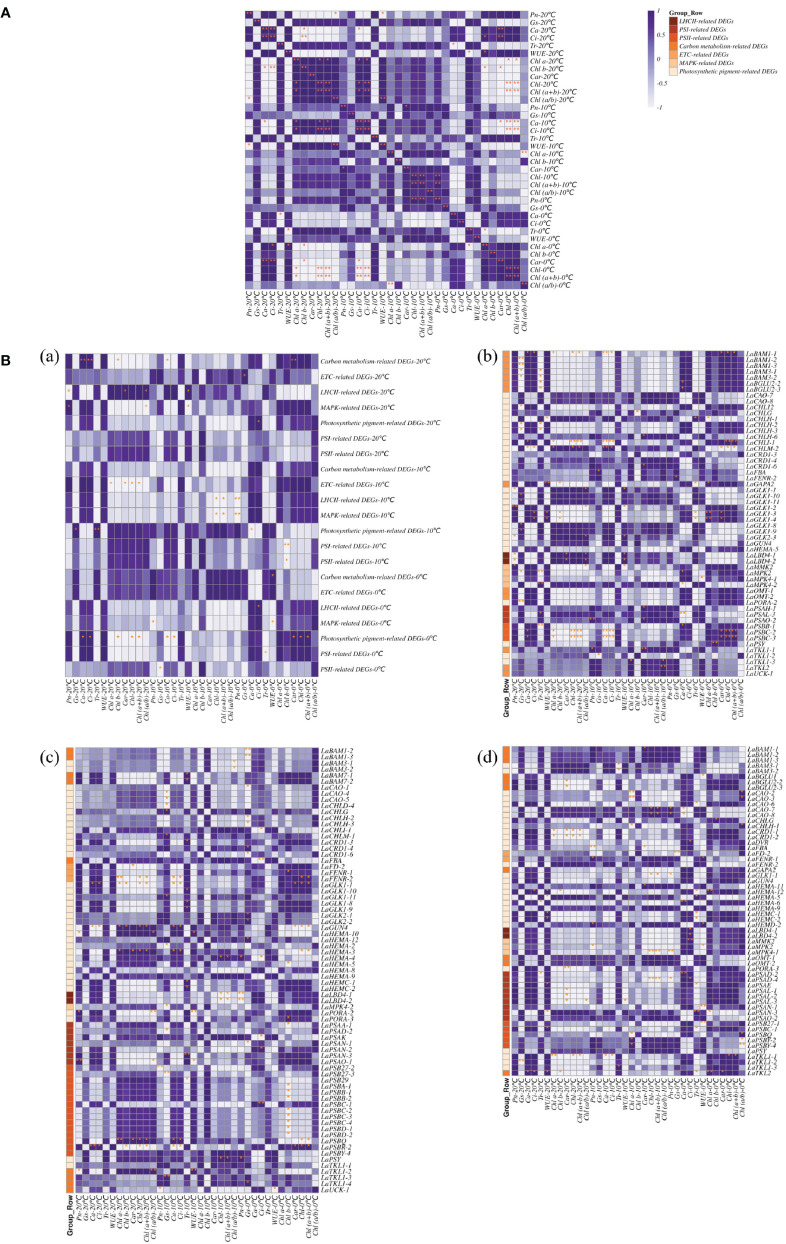
Correlation of photosynthesis related DEGs and photosynthetic physiological indexes in *L. angustifolia* leaves. **(A)** Correlation between photosynthetic physiological indexes in *L. angustifolia* leaves; **(B)** (a): Correlation between photosynthesis related DEGs and photosynthetic physiological indexes in *L. angustifolia* leaves. (b–d): Correlation between photosynthesis related DEGs and photosynthetic physiological indexes in *L. angustifolia* leaves at 20°C, 10°C, 0°C.

There were seven categories of related DEGs for LHCII, PSI, PSII, carbon metabolism, electron transport chain (ETC), MAPK, and photosynthetic pigment synthesis by classification on the functions of the proteins regulated by photosynthesis-related DEGs. Different correlation between photosynthetic physiological parameters and photosynthesis-related DEGs were revealed at different temperatures (**Figure 5A**).

At 20°C, significant negative correlation was observed between *P_n_* and *Chl (a+b)* (*p*<0.05), while *P_n_* was negatively correlated with LHCII-related DEGs (*p*<0.05) and positively correlated with MAPK-related DEGs (*p*<0.05), respectively. Highly significant positive correlations were observed between *C_a_* and *C_i_*, as well as between *C_a_*, *C_i_* and carbon metabolism-related DEGs (*p*<0.01) (**Figure 5B** (a, b)).

At 10°C, *P_n_* was found to be significantly positive with *Car* (*p*<0.05). Highly significant positive correlation exhibited between *Chl* and *Chl (a+b)* as well as between *C_a_* and *C_i_* (*p*<0.01), while *Chl* and *Chl (a+b)* both formed a significantly negative correlation MAPK-related DEGs (*p*<0.05) and LHCII-related DEGs (*p*<0.01) (**Figure 5B** (a, c)).

At 0°C, *T_r_* was negatively correlated with *Chl a* (*p*<0.05), negatively correlated with PSI-related DEGs (*p*<0.05).*Chl* was positively correlated with *Chl* (a+b) (*p*<0.01). Moreover, *Car*、*Chl* and *Chl (a+b)* were positively correlated with PSI-related DEGs (*p*<0.05) (**Figure 5B** (a, d)).

### 3.5 Amino acid sequence analysis of key DEGs for photosynthesis in *L. angustifolia* leaves

#### 3.5.1 Analysis of physicochemical properties of amino acid sequences

Based on the results of 3.4, 21 key DEGs correlating with photosynthetic indexes were selected for analysis (**Table 5**). Amino acid sequence analysis of *L. angustifolia* photosynthesis-associated key DEGs showed a range of variation in their amino acid length (101-756), molecular weight (11 386.51-81 769.67 kDa), and theoretical isoelectric point (4.48-9.94), indicating that the regulation of *L. angustifolia* photosynthesis-related proteins are distributed from acidic to basic. The grand average of hydropathicity was negative in 17, accounting for 96.92%, indicating there were hydrophobicity of the proteins in the mass related to the regulation of photosynthesis in *L. angustifolia*.

**Table 5:** Physical and chemical information of amino acid sequence of key DEGs of photosynthesis in *L. angustifolia* leaves.

Gene name	Number of amino acids	Molecular weight (kDa)	Theoretical pI	Grand average of hydropathicity (GRAVY)	Aliphatic index	Instability index	Subcellular localization
*LaPSAA*	505	56212.35	6.22	0.118	0.118	29.59	Plasma membrane
*LaPSBA*	352	38863.49	5.12	0.338	95.94	35.44	Plasma membrane
*LaPSBQ*	213	23948.47	9.54	-0.241	92.63	61.28	Cytoplasm
*LaBAM1-1*	414	46919.90	5.31	-0.473	68.60	35.59	Mitochondrion
*LaBAM1-2*	101	11386.51	9.94	0.162	96.53	56.62	Chloroplasts
*LaBAM1-3*	220	23116.26	7.76	-0.099	72.77	51.19	Cytoplasm
*LaBAM3-1*	179	19867.99	8.78	-0.235	81.12	30.78	Cytoplasm
*LaBAM3-2*	295	32939.02	7.65	-0.574	60.98	35.64	Mitochondrion
*LaFBA*	230	26883.41	4.48	-0.323	85.91	38.47	Cytoplasm
*LaFD-1*	146	16558.81	9.46	-0.505	66.16	62.88	Nucleus
*LaFENR-1*	273	30838.02	6.67	-0.638	67.47	39.65	Cytoplasm
*LaFENR-2*	186	21310.39	6.37	-0.547	72.31	43.55	Mitochondrion
*LaTKL1-1*	198	21298.11	8.01	-0.220	80.86	42.15	Mitochondrion
*LaTKL1-2*	756	81769.67	6.48	-0.194	85.12	38.44	Chloroplasts
*LaTKL1-3*	279	29959.43	6.97	-0.013	86.45	33.08	Cytoskeleton
*LaTKL2*	245	26730.65	5.11	-0.424	74.20	30.62	Cytoplasm
*LaMMK2*	246	27725.34	9.94	-0.056	102.20	34.47	Chloroplasts
*LaMPK4-1*	280	32593.22	5.28	-0.283	94.00	39.27	Cytoplasm
*LaPSY*	212	23794.41	9.35	-0.106	88.30	55.17	Nucleus
*LaGUN4*	250	28428.79	7.88	-0.614	63.68	40.66	Nucleus
*LaCHLG*	241	26299.47	8.62	0.107	104.81	27.34	Chloroplasts

The higher aliphatic index indicates the higher thermal stability of the protein, and the lower instability index indicates the higher overall stability of the protein. In *L. angustifolia*, LaCHLG protein has the highest thermal stability and overall stability, LaPSAA protein has the lowest thermal stability, and LaFD-1 protein has the lowest overall stability (**Table 5**). Subcellular localization of regulatory photosynthesis-related proteins were located in the cell membrane, cytoskeleton, cytoplasm, chloroplast, mitochondrion, nucleus, but mainly in the cytoplasm (**Table 5**).

#### 3.5.2 Analysis of conserved structural domains of amino acid sequences

**Figure 6:**
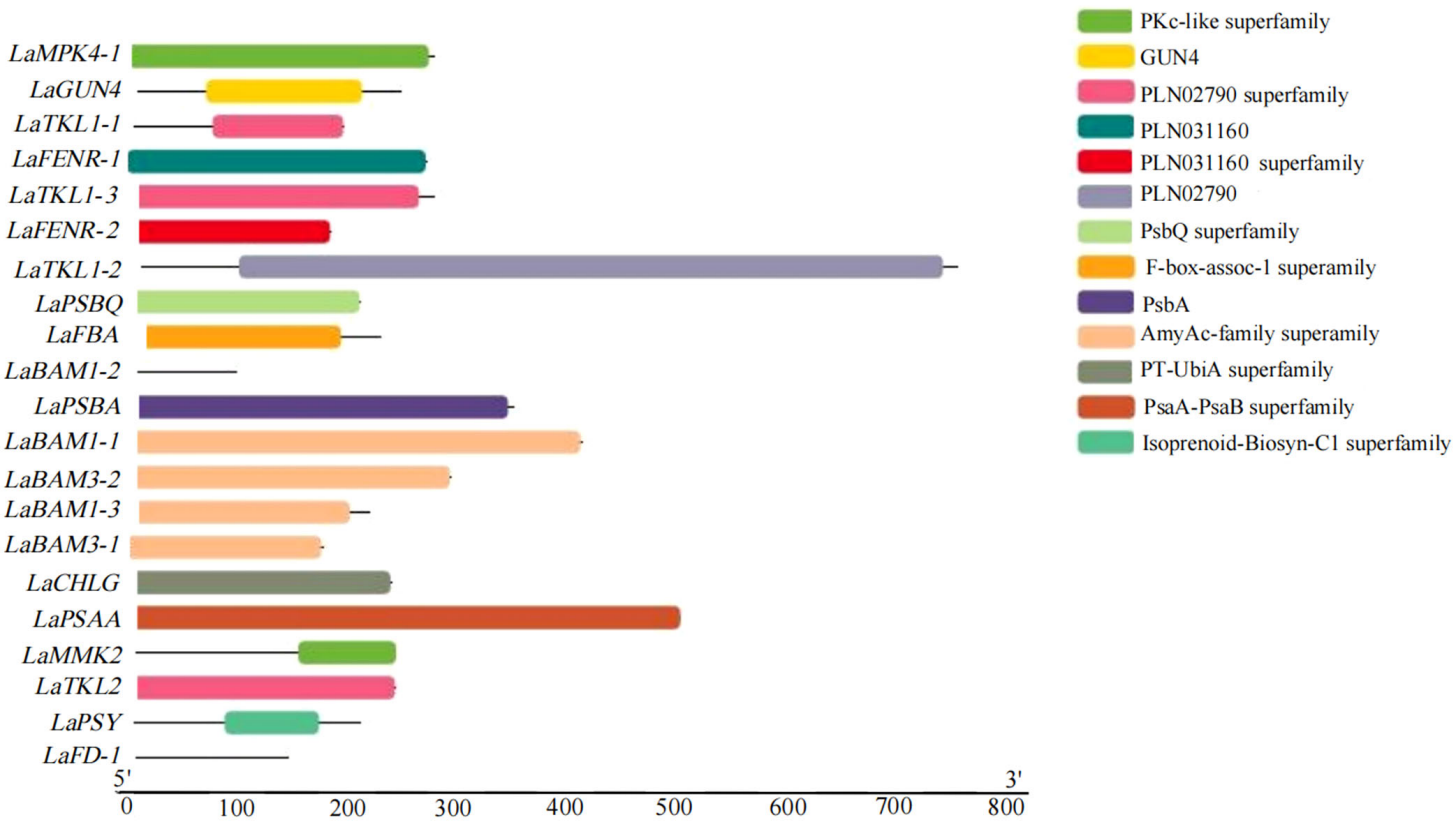
Visualization analysis of key DEGs conserved domains of photosynthesis in *L. angustifolia* leaves.

A total of 9 domains were obtained by analyzing the conserved domains (CDD) of amino acid sequences of 21 key DEGs for photosynthesis in *L. angustifolia*. The amino acid sequences encoded by LaBAM have the AmyAc-family superfamily domain, the amino acid sequences encoded by LaTKL1-1, 1-2, 1-3, and LaTKL2 have PLN02790, and the amino acid sequences encoded by LaGUN4, LaPSBQ, LaPSBA, and LaPSAA have their motifs-GUN4 domain, PsbQ superfamily domain, PsbA domain, and PsaA-PsaB superfamily domain (**Figure 6**).

#### 3.5.3 Predicted three-dimensional structures of key proteins for photosynthesis in *L. angustifolia* leaves

According to the 9 domains mentioned in 3.5.2, the 9 proteins were selected for structural prediction. The structures of key proteins for photosynthesis in *L. angustifolia* leaves were predicted by Phyre 2. Since LaPSAA, LaPSBA, LaPSBQ, LaBAM1-1, LaBAM3-2, LaFENR-1, LaTKL1-3, LaTKL2, LaMPK4-1, and LaGUN4 proteins were all highly consistent with template (Identity≥85%), implying the predictions were reliable.

**Figure 7:**
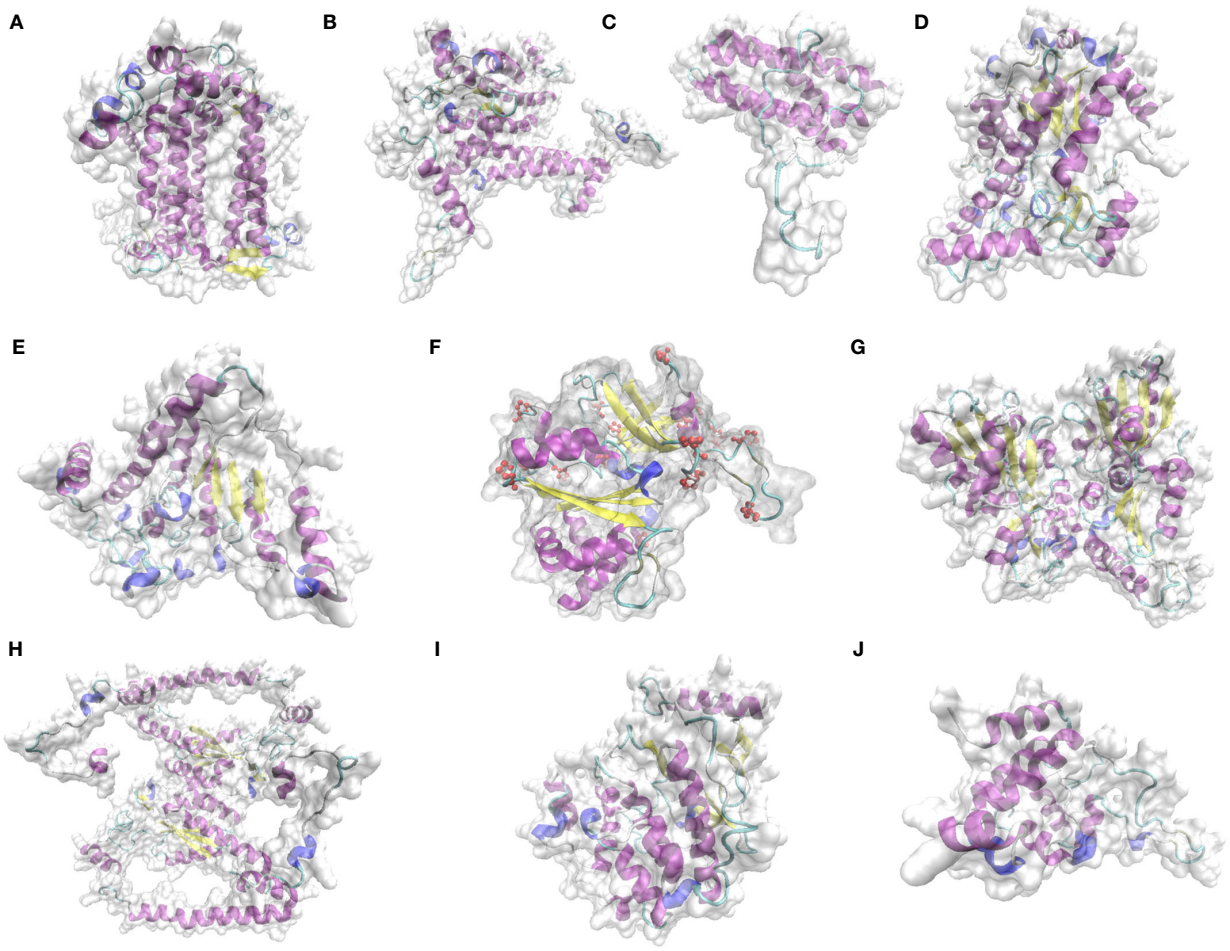
Prediction of key protein structure models of *L. angustifolia* leaves. **(A)**, LaPSAA protein; **(B)**, LaPSBA protein; **(C)**, LaPSBQ protein; **(D)**, LaBAM1-1 protein; **(E)**, LaBAM3-2 protein; **(F)**, LaFENR-1 protein; **(G)**, LaTKL1-3 protein; **(H)**, LaTKL2 protein; **(I)**, LaMPK4-1 protein; **(J)**, LaGUN4 protein. The yellow portion represents the β-fold, the purple portion represents the α-helix, the light blue portion represents the β-inflection, the dark blue portion represents other folds, red atoms represent proline, and the transparent area represents the complete structure of protein.

As shown in **Figure 7**, LaPSBQ protein only has α-helix and β-inflection, and LaGUN4 protein only has α-helix, β-inflection, and other folds. The remaining eight proteins have β-fold, α-helix, β-inflection, and other folds, and LaFENR-1 protein has proline (**Figure 7**). All 10 proteins have α-helices and β-inflections, indicating that the proteins are structurally stable with amino acid residues containing polar and charged amino acids (**Figure 7**).

### 3.6 Verification of differential gene expression in *L. angustifolia* leaves

**Figure 8:**
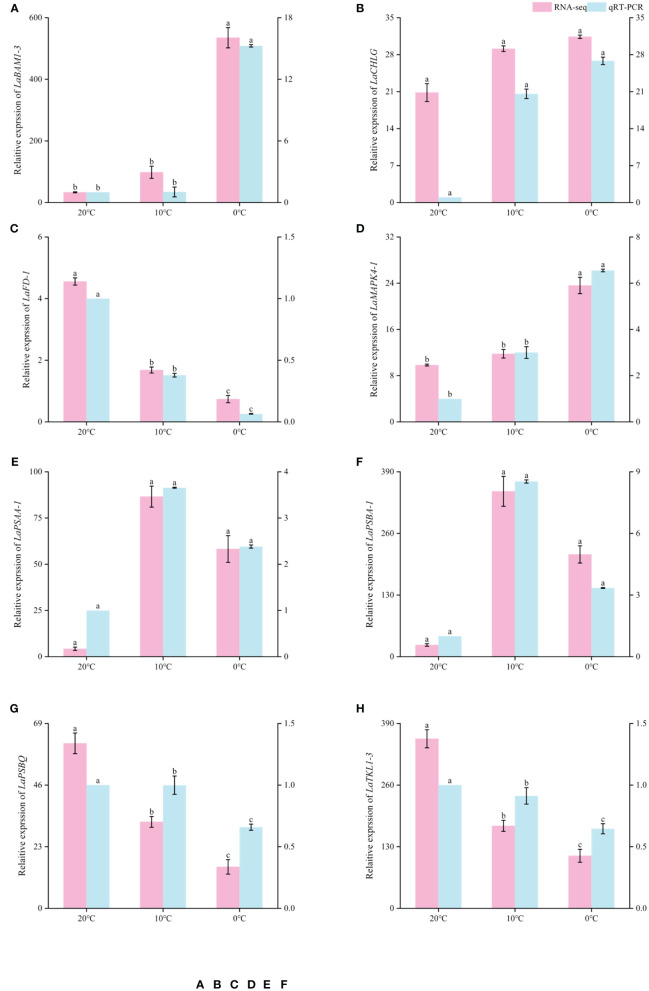
Comparison of RNA-seq and qRT-PCR expression levels of *L.angustifolia* leaves at different temperatures. The left Y-axis is the RNA-seq expression levels, and the right Y-axis is the qRT-PCR expression levels. Different letters indicate significant differences (*p*<0.05), the order of letters is based on the mean from largest to smallest. M ± S.E.(n=9).

In order to verify the reliability of RNA sequencing results, 8 DEGs were selected from the above research results and amplified by qRT-PCR, and their relative expression levels were calculated. qRT-PCR results showed that *LaBAM1-3*, *LaCHLG*, and *LaMAPK4-1* were relatively up-regulated with the decrease of temperature. *LaFD-1*, *LaPSBQ*, and *LaTKL1-3* were relatively down-regulated with the decrease of temperature (**Figure 8**). *LaPSAA-1*, and *LaPSBA-1* were increased at 0°C compared to 20°C (**Figure 8**). This result was basically consistent with the gene expression trend in transcriptome sequencing results, indicating that the sequencing results in this study were reliable, and the correlation analysis based on the sequencing results was objective (**Figure 8**).

## 4 Discussion

### 4.1 The light-response models responding to low temperature in *L. angustifolia*

In order to describe the light-response processes of plants, the most extensively utilized model is MRH, RH, NRH, and EXP (Li et al., 2019). In this study, the fitted values based on MRH are closest to the actual measured values in *L. angustifolia*’s leaves (**Table 2**), which could more accurately reflect the light-response processes under low temperature stress (**Figure 1**; **Table 2**). Therefore, photosynthetic parameters including *LCP*, *R_d_*, and *Φ* computed by this model could reveal the photosynthetic physiological characteristics when the plants were in response to low temperature.

*LCP* displayed an increasing trend if respiration proportion in total leaf CO_2_ exchange is high (Kaipiainen, 2009). In our research, *LCP* fitted by MRH decreasing at 10°C and increasing at 0°C illustrates that *L. angustifolia* performed photosynthesis to accumulate energy to combat the temperature drop (10°C), and then sustained life activities through respiration when exposed to low temperatures (0°C) (**Table 2**).

CO_2_ released by *R_d_* can be directly recycled in photosynthesis, which reduces the values of *R_d_* (Kang et al., 2014). In this study, *R_d_* of *L. angustifolia* showed a downward trend with the decrease of temperature, indicating that the ability to recycle CO_2_ released by *R_d_* increased during photosynthesis, which was consistent with the higher values of *C_i_* and *C_a_* compared to the control at low temperature (*p*<0.05) (**Tables 2**, **3**).

The apparent quantum efficiency (*Φ*) is a reflection of the light energy utilization efficiency of plants (Yao et al., 2017). In this study, the *Φ* of *L. angustifolia* leaves declined with the decreasing temperature, demonstrating that light energy utilization efficiency reduced due to the low temperature stress (**Table 2**). The PAR values were 1 728.75 ± 255.47 *μ*mol·m^-2^·s^-1^ in Harbin, northern China, from early growth stage to vigorous stage of *L. angustifolia*. Meanwhile, the fitting *LSP* was 1 500 *μ*mol·m^-2^·s^-1^ in the optimal MRH, indicating that *L. angustifolia* had high light adaptability in this area.

### 4.2 Photosynthetic physiological characteristics responding to low temperature in *L. angustifolia*

Chlorophyll degradation caused by low temperature stress seriously affected plant photosynthesis (Sachdev et al., 2023). In the present study, contents of *Chl a*, *Chl b*, *Car*, and *Chl* in *L. angustifolia* leaves decreased with the temperature dropping (**Table 3**), which might be the cause of the damage of pigment biosynthesis pathway or pigment degradation induced by low temperature stress. In chlorophyll biosynthesis pathway, chlorophyll synthesis is positively regulated by *CHLH* and *GUN4* (Kopečná et al., 2015; Shah et al., 2022), whereas CHLG, being a member of CHLase, is involved in degradative reactions of *Chl* by catalyzing *Chl a* into degreased *Chl a* (Shah et al., 2022). In the current study, GUN4 domain contained in *LaGUN4* functioned to regulate chlorophyll biosynthesis and intracellular signaling as porphyrin-binding protein (**Figure 6**). With the temperature decreasing, down-regulated expression of *LaCHLH-1*, *2*, *3* and *LaGUN4* and up-regulated expression of *LaCHLG* (**Figure 3B**) were molecular factors regulating the reduction of *Chl a*, *Chl b*, and *Chl* content (**Table 3**), which leads to the decrease of light energy collected by antenna system and light energy utilization rate (Wang et al., 2022). *Chl a* exists in both the core and LHC of the photosystem, but *Chl b* only exclusively exists in LHC (Khan et al., 2023). In this study, *Chl a/b* did not show significant differences at the three temperatures (*p*<0.05) (**Table 3**). Therefore, the changes of photosynthetic pigments in *L. angustifolia* adapted to low temperature stress need to be studied in depth.

*PSY* could participate in the regulation of carotene accumulation and chloroplast development in plants, and carotenoids could protect the photosynthetic apparatus against photo-oxidative damage caused by excessive light (Wang et al., 2020). This study showed a higher expression of *LaPSY* at 10°C than that at 20°C (**Figure 3B**), which presented a strategy to protect photosynthetic apparatus from photosynthetic oxidative damage by increasing carotenoids and to adapt to low temperatures in *L. angustifolia*. Coincidentally, the *LSP* fitted by MRH is close to the actual measured value at 10°C (**Table 2**), illustrating that *L. angustifolia* leaves are not damaged by light intensity under low temperature.

### 4.3 Molecular regulation of CO_2_ exchange responding to low temperature in *L. angustifolia*

A previous study has shown that BAM (β-amylase) is the main hydrolytic enzyme for starch degradation of *Arabidopsis* leaves, but only *AtBAM1* and *AtBAM3* are directly involved in starch degradation, playing a central role in the starch decomposition of leaves (Wang et al., 2023). Furthermore, *AaBAM3* overexpression increases freezing tolerance in *A. thaliana* by increasing soluble sugar content (Sun et al., 2021). In this study, *LaBAM3-1*, *2* with AmyAc-family super family domain was up-regulated with the decreasing temperature (**Figures 3A**, **6**), which was beneficial in accelerating starch degradation, increasing soluble sugar content, and improving the cold tolerance of *L. angustifolia*.

In previous research, plants lacking *BAM1* accumulated starch in guard cells and were impaired in stomatal opening, which implies an important role of *BAM1* in maintenance of stomatal conductance (Valerio et al., 2011). We found there was no significant differences in changes of stomatal conductance between the temperatures of 20°C, 10°C, and 0°C in *L. angustifolia* (**Table 3**). These phenomena were closely linked to the up-regulation of *LaBAM1-1*, *2*, *3* with the decreasing temperature (**Figure 3A**), enhancing the capability of stomatal adjustment and sustaining stomatal conductance at low temperatures (Sundas, 2021).

**Figure 9:**
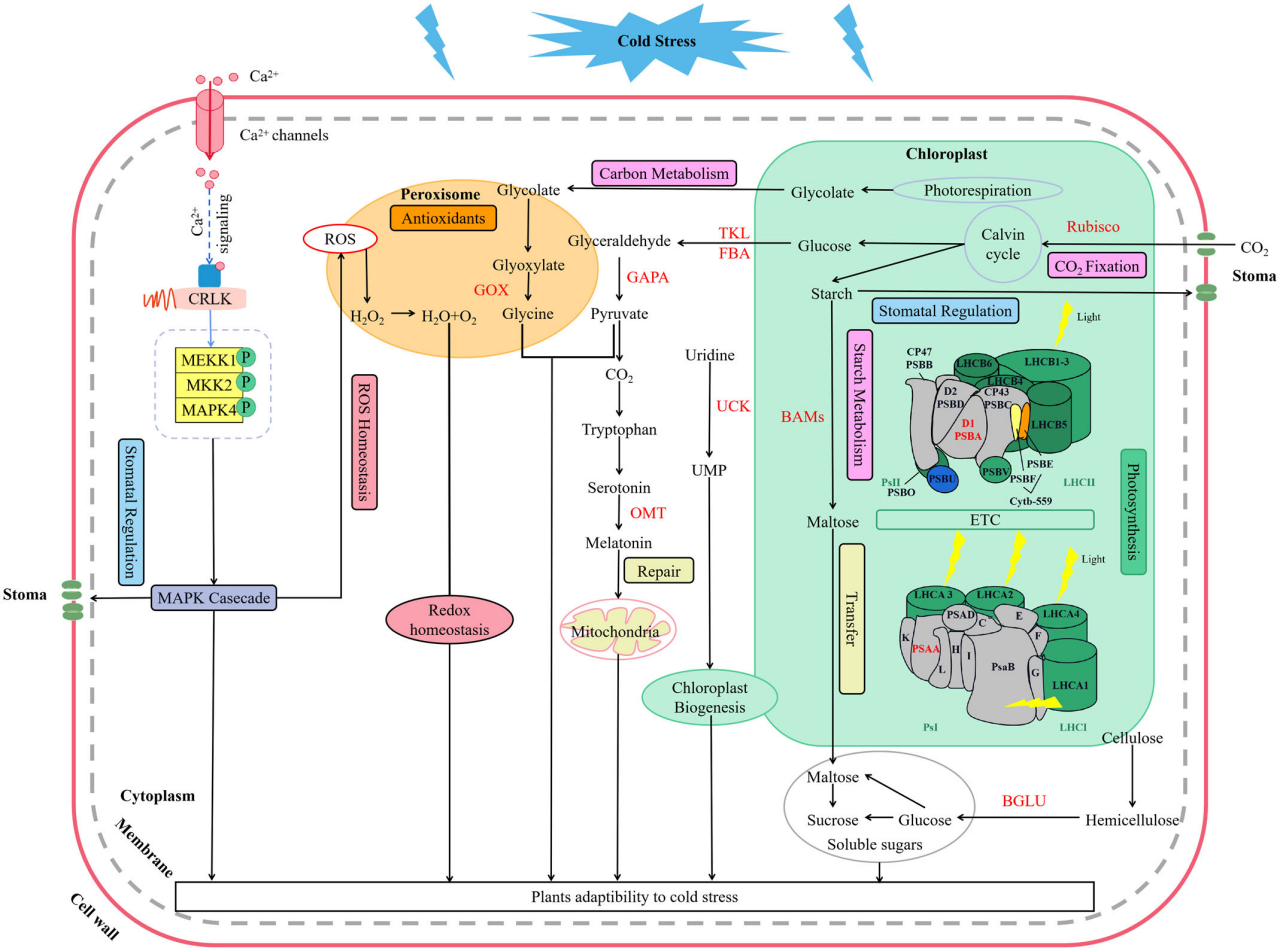
Key molecular regulation pathways of photosynthesis for *L. angustifolia* to adapt to low temperature. The red font in the figure refers to the key enzymes.

Low temperature could activate the ion channels on the membrane, which causes an instantaneous increase in cytosolic concentrations of Ca^2+^ (Yang et al., 2023). As a second messenger, Ca^2+^ activate MEKK1-AtMKK1/AtMKK2-AtMPK4 cascade for ROS homeostasis and stress tolerance as well (Liu and He, 2017) (**Figure 9**). Alterations of stomatal conductance responding to the mitogen-activated protein kinase (MAPK) cascade pathway triggered by cold stress could in turn regulate photosynthetic efficiency and photosystem response activity in *A. thaliana* (Liu and He, 2017) (**Figure 9**). In this study, it is appropriate that the up-regulation of *LaMPK4-1* and *LaMMK2* activated this cascade reaction, which was beneficial to the ROS steady state and led to the stomatal conductance decrease when the temperature dropping in *L. angustifolia* leaves (**Figure 3A**).

Under stress conditions, D1 protein (PSBA) can repair PSII cycle (Hajihashemi et al., 2018) and helps the degradation of misfolded proteins into peptides and amino acids for recycling of new defensive proteins (Sundas, 2021). In the present study, *LaPSBA* with PsbA domain (**Figure 6**) was up-regulated at 10°C and 0°C compared with 20°C (**Figure 3A**). It is predicted that *LaPSBA* played roles in repairing PSII cycle, promoting cell recycling of defensive proteins, and then enhancing plant stress resistance in *L. angustifolia* leaves.

### 4.4 Molecular regulation of electron transport responding to low temperature in *L. angustifolia*

PsaA is a crucial structural component of PSI in which FD1 is a ferredoxin-electron carrier in the electron transport chain, and FENR catalyzes reversible electron transport between ferredoxin and NADP in PSI (Mehmood et al., 2021). In *L.angustifolia*, *LaPSAA* has PsaA-PsaB superfamily domain (**Figure 6**) and the proteins with this domain are important enzymes for light-driven electron transport in PSI. Up-regulated expression of *LaPSAA* could be helpful in promoting photosynthetic electron transport with decreasing temperature, while trends of down-regulated expression of *LaFD-1*, *2* and *LaFENR-1*, *2* (**Figure 3A**) were similar to *A. thaliana* with no benefit to photosynthetic electron transport when the temperature decreased (Mehmood et al., 2021). The complex mechanisms of the electron transport need further study in *L. angustifolia*.

However, *LaUCK* was up-regulated with the decreasing temperature in *L. angustifolia* leaves (**Figure 3A**). It is known that UCK (uridine cytidine kinase) is involved in chloroplast biogenesis via the pyrimidine salvage pathway (Ohler et al., 2019), promoting the generation of UMP (uracil nucleotide), thus improving the ability of plants to adapt to low temperature stress (**Figures 3A**, **9**). Therefore, up-regulation of *LaUCK* had a positive effect on enhancing cold tolerance of *L. angustifolia.*

PsbQ, as one of PSII reaction center components in higher plants, has the function of maintaining the stability of the photosystem (Yi et al., 2006; Su et al., 2023). Removal of PsbQ caused by low temperature stress was showed to lead to electron transmission defects on both the oxidizing and reducing sides of the photosystem, resulting in structural damage of PSII (Bricker and Frankel, 2011). In the present study, the amino acid sequence encoded by *LaPSBQ* has PsbQ superfamily domain which is related to oxygen release in plants (**Figure 6**). In *L. angustifolia*, the expression of *LaPSBQ* was down-regulated (0°C) in photosynthetic pathway (**Figure 3A**), implying that PSII underwent photodamage at 0°C.

Photosynthetic rate is an important parameter for measuring the photosynthetic capacity of plants (Han et al., 2019), reflecting the ability of plants to convert light energy to organic matter. In our research, there are no significant differences in the photosynthetic rate between different temperatures (**Table 2**). The phenomena in which the rate of photosynthesis was not significantly impacted by low temperature is similar to the result of unaffected photosynthesis in *A. thaliana* with removal of the PsbQ protein (Yi et al., 2006). This phenomenon also implies that photosynthesis in *L. angustifolia* is regulated in various ways to better cope with low temperatures, as subsequently discussed in the regulation of carbon dioxide assimilation.

### 4.5 Carbon dioxide assimilation regulation responding to low temperature in *L. angustifolia*

Studies have shown that increasing Rubisco activity can increase the rate of photosynthetic carbon metabolism and protect plants from photoinhibition (Cavanagh et al., 2023; Chen T. et al., 2023). RCA is the enzyme necessary for Rubisco to be catalytically active (He et al., 2017) and SBPase is the key enzyme that influences the regeneration rate of RuBP, which controls the flow of carbon in the Calvin cycle (Ding et al., 2017). Increased SBPase activity and accelerated RuBP regeneration result in higher carbon source levels in the Calvin cycle and increased photosynthetic rate, which improve photosynthetic function of leaves and thus enhance plant tolerance to low temperatures (Wang, 2011). In *L. angustifolia*, interactions existed between RCA and SBPase, SBPase and Rubisco were advantageous for plants maintaining proper photosynthetic rate at low temperature with RuBP completing carboxylation under the action of Rubisco (**Figures 3A**, **9**).

Rubisco, in addition to its carboxylase activity, can also catalyze the oxygenation of RuBP (Aroca et al., 2023). The oxygenation of RuBP by the oxygenase activity of Rubisco produces a toxic metabolite, 2-phosphoglycolate (2PG), that must be detoxified (Aroca et al., 2023). TKL, GAPA and GOX are the key enzymes in photorespiration which is a complex metabolic pathway (Aroca et al., 2023). And the pathway is necessary to detoxify and recycle the metabolites generated by the oxygenating activity of Rubisco in plants (Roque et al., 2023). TKL (transketase) is involved in the photosynthetic carbon cycle (Nan et al., 2021) and GAPA (glyceraldehyde triphosphate dehydrogenase) have catalytic activity in the Calvin cycle as well as in the oxidative pentose phosphate pathway (PPP) (Yang et al., 2006). In *L. angustifolia*, expressions of *LaGAPA-2*, *3*, *LaTKL1-1*, *2*, *3*, *4* and *LaGOX* were down-regulated with temperature decreasing (**Figure 3A**), therefore, the photorespiration was inhibited. Photorespiration affects photosynthesis efficiency and one of its most prominent products is H_2_O_2_ (hydrogen peroxide) (Roque et al., 2023). Under conditions of suppressed photorespiration, the H_2_O_2_ level was high (García-Calderón et al., 2023), and then H_2_O_2_ can oxidize glyoxylate into CO_2_ (Ogren, 1984). In our study, the *C_i_* is higher at 0°C than at 20°C (**Table 3**), which is consistent with the above results. The increase of *C_i_* could enhance the photochemical activity of PSII, which increases the electron transfer on the receptor site of PSII (Ullah et al., 2023).

Moreover, *LaOMT-1*, *2* exhibited up-regulation with the decreasing temperature, and melatonin is synthesized under the action of OMT (o-methyltransferase) (**Figures 3A**, **9**). Melatonin functions in abiotic adaptation through repairing ruptured mitochondria and chloroplast in plants (Lee and Back, 2019; Tan and Reiter, 2019). FBA (fructose-1, 6-diphosphate aldolase) is involved in glycolysis (Qiu et al., 2023). The higher expression of FBA weakens carbohydrate metabolism and accumulates large amounts of sucrose during cold stress (Sundas, 2021). In this study, *LaFBA* expression was up-regulated with the decreasing temperature (**Figure 3A**), predicting that *LaFBA* could promote cold stress resistance considering sucrose accumulation in *L. angustifolia* at low temperature. Based on these findings, we conclude that photosynthesis-related genes could regulate the multiple sites of photosynthetic process and pigments to increase cold tolerance of *L. angustifolia* under low temperature stress.

## 5 Conclusions

In this work, we performed a comprehensive investigation of the mechanisms underlying *L.angustifolia* adaptability to cold stress at both the physiological and molecular levels. This study presents that MRH is the best fitting model for the *P_n_-*PAR response process under low temperature conditions and 1 500 *μ*mol·m^-2^·s^-1^ is the optimal photosynthetic effective radiation of *L. angustifolia* in Harbin, China. Cold decreased photosynthetic efficiency, while the values of *C_i_* and *C_a_* were significantly higher at 0°C and 10°C than those at 20°C (*p*<0.05), which increased the ability to recycle CO_2_ released during photosynthesis. In addition, a number of regulatory genes and important pathways contribute crucially to the high cold tolerance in *L. angustifolia*. As shown in **Figure 9**, cold signal is transducted rapidly via Ca^2+^ signaling and MAPK casecade, consequently regulating stomatal conductance, controlling CO_2_ transport. Following that, CO_2_ trigger a series of photosynthetic gene expressions (*LaBAM3-1*, *2*, *LaMMK2*, *LaMPK4*, and *LaFBA*), which could repair the PSII cycle to protect photosynthetic organs from damage, thereby increasing soluble sugar accumulation, improving cold resistant of *L. angustifolia*. Meanwhile, *L. angustifolia* can regulate the expressions of *LaPSY*, *LaGAPA-2*, *3*, *LaGOX*, and *LaTKL1-1*, *2*, *3*, *4* to control the chloroplast biogenesis, remove excess ROS from the plant and repair ruptured mitochondria, ultimately enhancing the plant cold tolerance. Taken together, we found the key molecular regulation pathways of photosynthesis for *L. angustifolia* to adapt to low temperature, which elucidates the internal regulatory mechanisms of high cold resistance in *L. angustifolia*.

## Data availability statement

The datasets presented in this study can be found in online repositories. The names of the repository/repositories and accession number(s) can be found below: https://www.ncbi.nlm.nih.gov/,PRJNA765132.

## Author contributions

LL: Writing – original draft, Writing – review & editing. YucL: Writing – review & editing, Writing – original draft. YinL: Writing – review & editing. ZS: Writing – review & editing. YunL: Writing – review & editing. ZY: Funding acquisition, Writing – review & editing. CF: Writing – review & editing.
