# Corrigendum: Transcriptome analyses reveal photosynthesis-related genes involved in photosynthetic regulation under low temperature stress in *Lavandula angustifolia* Mill.

**DOI:** 10.3389/fpls.2023.1339454

**Published:** 2023-12-01

**Authors:** Ling Li, Yuchen Liang, Yinan Liu, Zeyi Sun, Yuning Liu, Zening Yuan, Chang Fu

**Affiliations:** ^1^ Key Laboratory of Aquatic Biodiversity Research in Hei Longjiang Province, Harbin Normal University, Harbin, China; ^2^ Heilongjiang Provincial Key Laboratory of Plant Biology in Ordinary Colleges and Universities, Harbin Normal University, Harbin, China; ^3^ College of Science and Technology, Harbin Normal University, Harbin, China

**Keywords:** DEGs (differentially expressed genes), *Lavandula angustifolia* Mill., low temperature stress, photosynthetic regulation, qRT-PCR

**Incorrect Funding**

In the published article, there was an error in the Funding statement. Due to our mistakes, the fundings number were wrong.

The Funding statement previously stated:

“The author(s) declare financial support was received for the research, authorship, and/or publication of this article. The research was financially supported by Graduate innovation research project of Harbin Normal University(HSDSSCX2021-02 and HSDSSCX2021-10) and Digital economy, bio-economy, carbon peaking and carbon neutrality strategy, and creative design special projects of Harbin Normal University (XGT202307).”

The corrected Funding statement appears below.

“The research was financially supported by Graduate innovation research project of Harbin Normal University (HSDSSCX2021-02 and HSDSSCX2022-35) and Digital economy, bio-economy, carbon peaking and carbon neutrality strategy, and creative design special projects of Harbin Normal University (XGT202307).”

The authors apologize for this error and state that this does not change the scientific conclusions of the article in any way. The original article has been updated.

